# Supplementary material for: Content-rich biological network constructed by mining PubMed abstracts
Source: BMC Bioinformatics. 2004 Oct 8;5:147. doi: 10.1186/1471-2105-5-147 (PMC528731; doi:10.1186/1471-2105-5-147)
Supplement: Additional File 2 — The original results of the above study (non-essential files are deleted to keep the file size under the limit set by BMC bioinformatics). [file 1471-2105-5-147-S2.bz2 › chilibotAdditionalFile2/dip20/126ID9261098E470/html/RAF1_HRAS.html]

 


 **RAF1** and **HRAS** 
  
Found 26 abstracts in PubMed, retrieved 20.  
 

 What does Google say? 
 PDF only 
| .edu only 

---

**Interactive relationship** (e.g. stimulation, inhibition, etc)

**Inhibitory relationship**- Furthermore, we identified common and distinct targets in cells transformed by mutant  **HRAS** , KRAS and NRAS, as well as 61 putative target genes controlled by the RAF  [ **RAF1** ]  MEK ERK pathway in reverted cells treated with the MEK specific inhibitor PD 98059.  Ref: 10655059 Nat Genet, 2000
**Neutral relationship**- Treatment of human Raf 1  [ **RAF1** ]  purified from insect Sf9 cells co expressing c H Ras  [ **HRAS** ]  and Src Y527F in which phenylalanine replaces tyrosine at residue 527 with either serine threonine or tyrosine phosphatases resulted in enzymatic inactivation of Raf 1  [ **RAF1** ] .  Ref: 7604263 Science, 1995
- Using genomic microarray, we detected oncogene copy number gains of  **RAF1**  3p25, CTSB 8p22, PAK1 11q13, and JUNB 19p13 in 5 of 7 cases of mycosis fungoides MF Sezary syndrome SS 71%, gains of FGFR1 8p11, PTPN 20q13, and BCR 22q11 in 4 cases 57%, and gains of MYCL1 1p34, PIK3CA 3q26,  **HRAS**  11p15, MYBL2 20q13, and ZNF217 20q13 in 3 cases 43%.  Ref: 12393503 Blood, 2003

**Non-interactive relationship** (e.g. studied together, co-existance, homology, etc.)

- Finally, a dominant inhibitory form of RalA suppresses the transforming activities of both RasH  [ **HRAS** ]  and Raf  [ **RAF1** ] .  Ref: 8631302 EMBO J, 1996
- There was a good correlation between increasing ODC specific activity and change in morphology from normal to transformed in the nu Raf  [ **RAF1** ] ,  **HRas**  61L, and RhoA 63L clones.  Ref: 9661886 Cancer Res, 1998
- These results suggest that transformation by the c H ras  [ **HRAS** ]  oncogene is not mediated by the activation of raf  [ **RAF1** ]  protein kinase.  Ref: 1995648 J Biol Chem, 1991
- Northern blot analyses using 11 different oncogene probes revealed that 5 oncogenes, i.e. c myc, c H ras  [ **HRAS** ] , c sis, c raf  [ **RAF1** ] , and c fos, were expressed.  Ref: 8131089 Cancer Invest, 1994
- The genes chosen for analysis included both cytoplasmic c raf  [ **RAF1** ]  1, rasH  [ **HRAS** ] , rasK, and rasN and nuclear c fos and c myc proto oncogenes encoding proteins involved in the transduction of signals from protein tyrosine kinase growth factor receptors.  Ref: 8507485 Mol Reprod Dev, 1993
- After four or eight weeks, the lung tissue of the mice treated with NNK displayed a significantly high level of expression in c myc, c raf  [ **RAF1** ] , and c H ras  [ **HRAS** ]  oncogenes, and they were all inhibited by tea drinking with inhibitory rates of 50%, 20%, and 50%, respectively.  Ref: 8584456 Nutr Cancer, 1995
- Furthermore, although constitutively activated Ra1A does not induce oncogenic transformation on its own, its expression enhances the transforming activities of both RasH  [ **HRAS** ]  and Raf  [ **RAF1** ] .  Ref: 8631302 EMBO J, 1996
- Moreover, as revealed in experiments with growing cultures, neither transformation by the c H ras  [ **HRAS** ]  oncogene nor reversion from the transformed state altered the electrophoretic mobility of the raf  [ **RAF1** ]  protein or the level of its actin kinase activity.  Ref: 1995648 J Biol Chem, 1991
- Apoptosis and cell proliferation were determined by immunocytological techniques and the markers p53, p21WAF Cip, Bax, Bak, Bcl 2, cyclin D1 and PCNA, and also screened for signal transduction indicators such as c H ras  [ **HRAS** ] , c fos and raf 1  [ **RAF1** ] .  Ref: 9494580 Anticancer Res< MedlineTA>Anticancer Res, 1991
- Introduction of recombinant phage DNA into established 208F and Rat 2 cells and into  **HRAS** , v fgr, v fms and v raf  [ **RAF1** ]  transformed rat cell lines resulted in inhibition of growth and induction of programmed cell death.  Ref: 8108134 Oncogene, 1994
- Signal transducers included phospholipase C gamma PLC gamma, protein kinase C gamma PKC gamma, and two proto oncogenes, c H ras  [ **HRAS** ]  and c raf  [ **RAF1** ]  1.  Ref: 8361757 Oncogene, 1993
- DNA probes for the NRAS,  **HRAS** , KRAS2, LCK,  **RAF1** , MET, MYCL1, MYCN, MYB, ERBB2, FOS, CSF1R, and SRC protooncogene loci.  Ref: 8095010 Cytogenet Cell Genet, 1993
